# Exploring the value of blood urea nitrogen-to-albumin ratio in patients with acute pancreatitis admitted to the intensive care unit: a retrospective cohort study

**DOI:** 10.3389/fnut.2025.1435356

**Published:** 2025-04-16

**Authors:** Jianjun Wang, Han Li, Pei Yang, Xi Chen, Sirui Chen, Lan Deng, Xintao Zeng, Huiwen Luo, Dongqing Zhang, Xianfu Cai, Hua Luo, Decai Wang

**Affiliations:** ^1^Department of Hepatobiliary Surgery, Mianyang Central Hospital, School of Medicine, University of Electronic Science and Technology of China, Mianyang, China; ^2^NHC Key Laboratory of Nuclear Technology Medical Transformation, Mianyang Central Hospital, School of Medicine, University of Electronic Science and Technology of China, Mianyang, China; ^3^Department of Cardiology, The Fifth Hospital of Wuhan, Wuhan, China; ^4^Department of Rehabilitation Medicine, Mianyang Central Hospital, School of Medicine, University of Electronic Science and Technology of China, Mianyang, China; ^5^Department of Urology, Mianyang Central Hospital, School of Medicine, University of Electronic Science and Technology of China, Mianyang, China

**Keywords:** acute pancreatitis, blood urea nitrogen-to-albumin ratio, all-cause mortality, MIMIC-IV, a cohort study

## Abstract

**Background:**

Although blood urea nitrogen and albumin alone are well-known clinical indicators, combining them as the blood urea nitrogen-to-albumin ratio (BAR) may provide additional prognostic information because they reflect the complex interplay between renal function, nutritional status, and systemic inflammation—all of which are key factors in the pathogenesis of acute pancreatitis (AP). Therefore, the objective of this study was to investigate the relationships between BAR and short- and long-term all-cause mortality (ACM) in patients with AP and to assess the prognostic significance of the BAR in AP.

**Methods:**

This retrospective investigation utilized information extracted from the Medical Information Mart for Intensive Care-IV (MIMIC-IV, Version 2.2) database. BAR was calculated using the BUN/ALB ratio obtained from the first measurement within 24 h of admission. R software was used to identify the optimal threshold for the BAR. The Kaplan–Meier (K–M) analysis was performed to compare mortality between the two groups. Multivariate Cox proportional hazards regression models and restricted cubic splines (RCS) were used to evaluate the association between BAR and 14-day, 28-day, 90-day, and 1-year ACM. The receiver operating characteristic curves were used to investigate the predictive ability, sensitivity, specificity, and area under the curve (AUC) of the BAR for short- and long-term mortality in AP patients. Subgroup analysis was performed to illustrate the reliability of our findings.

**Results:**

This study comprised a total of 569 patients. The R software determined the optimal threshold for the BAR to be 16.92. The K–M analysis indicated a notable rise in ACM in patients with higher BAR (all log-rank *p* < 0.001). Cox proportional hazard regression models revealed independent associations between higher BAR and ACM before and after adjusting for confounding variables at days 14, 28, 90, and 1 year. The RCS analysis revealed J-shaped correlations between the BAR and short- and long-term ACM. The AUCs of the BAR for predicting ACM at days 14, 28, 90, and 1 year were 73.23, 76.14, 73.49, and 71.00%, respectively, which were superior to those of BUN, ALB, creatinine, Sequential Organ Failure Assessment, and Acute Physiology and Chronic Health Evaluation-II. Subgroup analyses revealed no significant interaction between BAR and the vast majority of subgroups.

**Conclusion:**

This study revealed, for the first time, the unique prognostic value of BAR in ICU-managed AP patients. Higher levels of BAR were associated with higher short- and long-term ACM in ICU-managed AP patients.

## 1 Introduction

Acute pancreatitis (AP) is a globally prevalent gastrointestinal disease characterized by the premature activation of a variety of digestive enzymes, leading to self-digestion by the pancreas; most patients with AP experience abdominal pain as the first symptom (1). AP imposes a heavy burden on the healthcare system, with a reported annual incidence of approximately 34 cases per 100,000 people and an associated mortality rate of approximately 1–5% (2). Approximately 20% of patients with AP are severely ill, often experiencing dysfunction of other organs and systems, and require hospitalization in the intensive care unit (ICU) (3). Despite tremendous progress that has been made in the management of intensive care, the mortality rate of patients with severe AP remains high, and this population tends to face a higher number of complications, including pancreatic necrosis, pancreatic pseudocysts, and chronic pancreatitis (4). Therefore, the identification of robust prognostic indicators for stratifying high-risk individuals with adverse outcomes holds pivotal clinical significance.

In recent years, the role of some common laboratory parameters, such as calcitoninogen, C-reactive protein, blood urea nitrogen (BUN), albumin (ALB), creatinine (Crea), and serum calcium, has been investigated in predicting the prognosis of patients with AP (5). For example, BUN primarily reflects changes in renal function and protein metabolism, as well as subtle variations in cardiac output and neurohumoral activity. Elevated BUN levels are associated with poor prognosis in a variety of diseases, including heart failure, renal disease, and other critical illnesses, and similar associations have been found in critically ill AP patients (6, 7). ALB primarily reflects the nutritional status of the body and plays important physiological roles, including maintenance of osmotic balance, promotion of molecular transport, antioxidant and anti-inflammatory effects, and stabilization of vascular endothelial function (8). While hypoalbuminemia is a marker of malnutrition, systemic inflammation, and abnormal liver function (8), all of which can severely affect the prognosis of patients with AP. However, although BUN and ALB are valuable for prognostication in patients with AP, their use as a combination of blood urea nitrogen-to-albumin ratio (BAR) may provide additional prognostic information because together they reflect a complex interplay between renal function, nutritional status, and systemic inflammation, all of which are key factors in the pathogenesis of AP. Prior research has established a robust correlation between BAR and various medical conditions, including pneumonia, sepsis, chronic obstructive pulmonary disease, cancer, gastrointestinal bleeding, and cardiovascular disease (9–14). However, its specific role in AP has not been fully explored, especially in patients hospitalized in the ICU. Given the high mortality rate in ICU-managed AP patients and the need for reliable and easy-to-use prognostic markers, we hypothesized that BAR could serve as a practical and easily calculated marker to help clinicians assess the short- and long-term risk of death in these patients.

Hence, the objective of this investigation was to examine the correlation between BAR and prognosis among AP patients, utilizing the Medical Information Mart for Intensive Care-IV database (MIMIC-IV, version 2.2). Specifically, we assessed the relationships between the BAR and short- and long-term all-cause mortality (ACM) in AP patients, as well as the predictive value of the BAR for short- and long-term ACM within this cohort. By elucidating the prognostic value of the BAR in AP, our results may contribute to early risk stratification and optimization of clinical management strategies, ultimately improving patient prognosis.

## 2 Methods

### 2.1 Data source

The data utilized in this study were sourced from the MIMIC-IV (v 2.2) database. This database is a comprehensive repository maintained by the Massachusetts Institute of Technology Computational Physiology Laboratory, providing public access. It encompasses the medical records of all patients admitted to the Beth Israel Deaconess Medical Center (15). For the protection of patient confidentiality, all personal data underwent de-identification, and randomized codes were assigned in place of patient identifiers. As a result, this study did not necessitate informed consent or ethical approval. The research team underwent training through the Collaborative Institutional Training Initiative and completed the “Conflict of Interest” and “Study Data or Specimens Only” exams, thereby gaining access to the database. Remarkably, several validation procedures were used to guarantee the precision of the extracted data, including independent reviews of critical data points, consistency assessments, and the utilization of statistical software to detect and rectify potential input errors or disparities.

### 2.2 Study population

**Figure 1:**
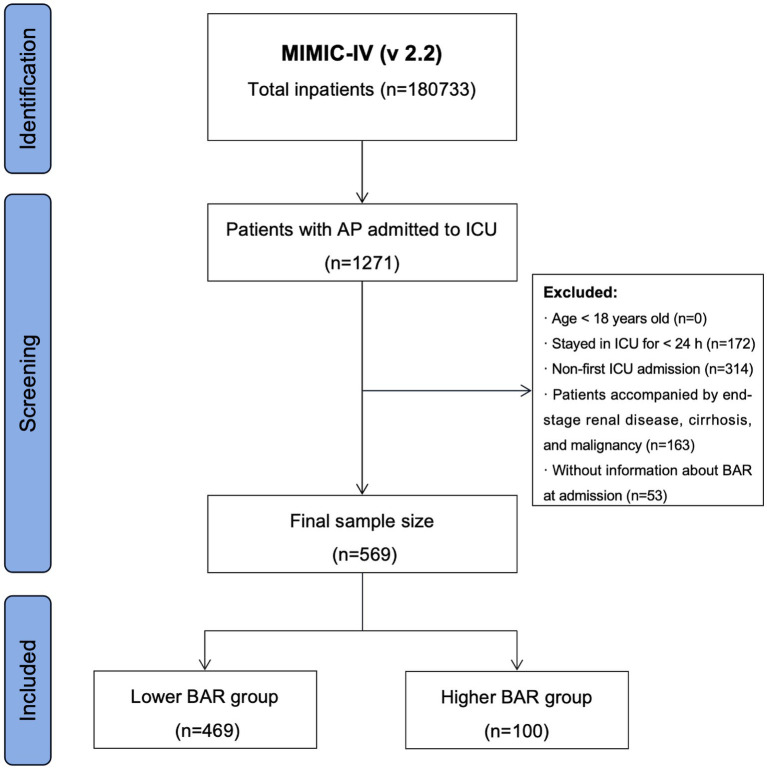
Flowchart for participants in MIMIV-IV (v 2.2).

Based on the International Classification of Diseases, Revision 9 (ICD-9) code 577.0 and International Classification of Diseases, Revision 10 (ICD-10) code K85–K85.92, hospitalization records for all AP patients were extracted from the MIMIC-IV (v 2.2) database. Strict exclusion criteria were applied to ensure the accuracy and robustness of the study results, as follows: (1) patients under 18 years old at their initial admission; (2) patients with recurrent ICU admissions for AP, with only data from their first admission retained; (3) patients with end-stage renal disease, cirrhosis, or malignant tumors; (4) patients with ICU stays of less than 24 h; and (5) patients for whom no information on BUN and/or ALB were recorded within 24 h of admission. Finally, this study included 569 patients (Figure 1).

### 2.3 Data extraction

The data extraction tools included PostgresSQL (version 13.7.2), Navicate Premium (version 16), and Structured Query Language (SQL). Data were extracted from the following major domains in this study: demographic variables, vital signs, clinical treatments, comorbidities, laboratory results, and clinical outcomes. Table 1 presents more details on data extraction.

**Table 1:** Covariates extracted in detail.

Items	Composition
Demographic variables	Age, Gender, Ethnicity
Vital Signs	HR, SBP, DBP, MAP, RR, SpO2, Temperature
Clinical Treatments	Vasopressin, Octreotide, Statins, Betablockers, Ventilation, CRRT, ERCP
Comorbidities	AKI, Sepsis, RF, HF, AF, Hypertension, Diabetes, Obesity
Laboratory variables	RBC, WBC, RDW, PLT, Hb, HCT, Crea, ALB, BUN, TBil, AST, ALT, GLU, PT, INR, Blood lipase, K, Na, TCa, AG, LAC
Clinical Outcomes	14-day ACM, 28-day ACM, 90-day ACM, 1-year ACM

HR, heart rate; SBP, systolic blood pressure; DBP, diastolic blood pressure; MAP, mean arterial pressure; RR, respiratory rate; CRRT, continuous renal replacement therapy; ERCP, endoscopic retrograde cholangiopancreatography; AKI, acute kidney injury; RF, respiratory failure; HF, heart failure; AF, atrial fibrillation; RBC, red blood cell; WBC, white blood cell; RDW, erythrocyte distribution width; PLT, platelet; Hb, hemoglobin; HCT, hematocrit; Crea, creatinine; ALB, albumin; BUN, blood urea nitrogen; TBil, total bilirubin; AST, aspartate aminotransferase; ALT, alanine aminotransferase; GLU, Glucose; PT, prothrombin time; INR, international normalized ratio; K, serum potassium; Na, serum sodium; TCa, serum total calcium; AG, anion gap; LAC, lactate; ACM, all-cause mortality.

### 2.4 Handling of abnormal and missing values

Using the STATA winsor2 command, the outlier variables were processed using the winsorization method at the 1 and 99% cutoffs. The research team used multiple estimation methods to address missing values. Variables with >15% of the values missing, such as height, C-reactive protein levels, and procalcitonin levels, were excluded.

### 2.5 Outcome indicators

In this study, the endpoints consisted of ACM at 14-, 28-, and 90-day, as well as 1-year post-admission, among patients diagnosed with AP.

### 2.6 Statistical analysis

Continuous variables were expressed as means ± standard deviation (SD) for normally distributed data and as medians (interquartile range [IQR]) for skewed distributions. Normally distributed continuous variables were analyzed using the t-test or analysis of variance (ANOVA), whereas skewed variables were analyzed using the Mann–Whitney U-test or Kruskal–Wallis test. Categorical data were expressed as numbers (%) and were compared using either the chi-square test or Fisher’s exact test. The optimal cutoff point for BAR in this study was determined using R software (version 4.3.2; Supplementary Figure 1). According to the determined optimal BAR cutoff, the study cohort was segregated into two cohorts: lower BAR and higher BAR. The Cox proportional hazards models were used to assess the association between BAR and study endpoints, yielding hazard ratio (HR) and 95% confidence interval (CI). Three models were utilized to adjust for confounding factors: Model 1 (baseline model), Model 2 (adjusted for age, sex, and ethnicity), and Model 3 (adjusted for age, sex, ethnicity, hypertension, diabetes, heart failure, respiratory failure (RF), sepsis, white blood cell count, platelet count, Crea level, vasopressin level, continuous renal replacement therapy (CRRT), and Sequential Organ Failure Assessment [SOFA]). The Kaplan–Meier (K–M) survival analysis was used to evaluate ACM across the two groups, with the log-rank test utilized to compare the survival curves. The restricted cubic spline (RCS) curves were used to investigate potential dose–response associations between BAR and ACM at different time points. The receiver operating characteristic (ROC) analyses were used to evaluate the predictive performance of BAR, BUN, ALB, Crea, SOFA, and Acute Physiology and Chronic Health Evaluation-II (APACHE-II) for ACM at different time points, and the area under the curve (AUC) was calculated. Finally, subgroup analyses were conducted to examine the consistency of BAR prognostic values across various subgroups. The subgroups were defined by age, sex, hypertension, diabetes, and RF. All analyses required a significance threshold of *p* < 0.05 (two-tailed). Data analysis was conducted using R software (version 4.3.2), STATA software (version 16.0), and IBM SPSS software (version 22.0).

## 3 Results

### 3.1 Baseline characteristics of the participants

Following stringent inclusion and exclusion criteria, a total of 569 patients diagnosed with AP were incorporated into this study (Figure 1). According to the identified optimal cutoff for BAR, participants were categorized into lower BAR (<16.92) and higher BAR (≥16.92) groups. Table 2 displays the baseline characteristics of the patients. Patients with higher BAR were usually older, with higher prevalences of acute kidney injury (AKI), sepsis, and RF. Red blood cell counts, hemoglobin levels, ALB levels, and total serum calcium levels were lower in patients with higher BAR compared to those with lower BAR, whereas the red blood cell distribution widths, anion gap levels, blood glucose levels, Crea levels, BUN levels, prothrombin times, and serum potassium ion levels were higher in the higher BAR group. Moreover, patients in the higher BAR group had higher ACM, as follows: 14-day mortality (5.33% vs. 21.00%, *p* < 0.001), 28-day mortality (7.89% vs. 32.00%, *p* < 0.001), 90-day mortality (13.65% vs. 43.00%, *p* < 0.001), and 1-year mortality (18.34% vs. 48.00%, *p* < 0.001).

**Table 2:** Baseline characteristics in patients with acute pancreatitis.

Variable	Overall (*n* = 569)	Lower BAR (<16.92, *n* = 469)	Higher BAR (≥16.92, *n* = 100)	*p* value
BAR	7.3 (4.2–12.9)	6.2 (3.8–9.2)	23.6 (20.6–29.8)	<0.001
Demographics				
Age, years	59 (46–73)	56 (45–71)	66 (57–77)	<0.001
Men, n (%)	327 (57.47)	268 (57.14)	59 (59.00)	0.73
Ethnicity, n (%)				0.79
Asian	21 (3.69)	17 (3.62)	4 (4.00)	
White	352 (61.86)	292 (62.26)	60 (60.00)	
Black	44 (7.73)	38 (8.10)	6 (6.00)	
Others	152 (26.71)	122 (26.01)	30 (30.00)	
Comorbidities				
Acute kidney injury, n (%)	405 (71.18)	321 (68.44)	84 (84.00)	0.002
Sepsis, n (%)	426 (74.87)	339 (72.28)	87 (87.00)	0.002
Respiratory failure, n (%)	258 (45.34)	197 (42.00)	61 (61.00)	<0.001
Heart failure, n (%)	98 (17.22)	75 (15.99)	23 (23.00)	0.09
Atrial fibrillation, n (%)	127 (22.32)	101 (21.54)	26 (26.00)	0.33
Hypertension, n (%)	278 (48.86)	239 (50.96)	39 (39.00)	0.03
Diabetes, n (%)	180 (31.63)	143 (30.49)	37 (37.00)	0.20
Obesity, n (%)	75 (13.18)	65 (13.86)	10 (10.00)	0.30
Vital sign				
Heart rate, beats/min	101 (84–117)	101 (85–118)	97.5 (82–112)	0.12
SBP, mmHg	126 (108–144)	129 (110–147)	118.5 (102–138.5)	0.002
DBP, mmHg	72 (59–86)	74 (61–89)	62 (52–72)	<0.001
MAP, mmHg	90.5 (77.7–103.7)	92.3 (78.7–106.3)	81.3 (73–92.7)	<0.001
RR, times/min	21 (17–25)	21 (17–25)	21.5 (16.5–26)	0.88
SPO_2_, %	96 (94–99)	96 (94–99)	97 (94.5–100)	0.06
Temperature, °C	36.8 (36.4–37.3)	36.9 (36.5–37.4)	36.5 (36.2–36.9)	<0.001
Laboratory parameters				
RBC	3.7 (3.2–4.2)	3.7 (3.2–4.2)	3.4 (3.0–4.1)	0.02
WBC	13.0 (9.0–18.6)	12.9 (8.9–18.5)	14.5 (9.4–19.5)	0.30
RDW	14.5 (13.6–15.8)	14.4 (13.6–15.7)	15.2 (14.25–16.2)	0.001
PLT	187 (130–271)	190 (130–275)	176.0 (128.5–241.5)	0.30
Hb	11.2 (9.7–13.0)	11.3 (9.8–13.0)	10.7 (9.2–12.6)	0.02
HCT	34.0 (29.4–38.9)	34.3 (29.7–39.0)	32.3 (28.6–38.1)	0.06
AG	14 (12–17)	14 (12–16)	16.0 (12.5–20.5)	<0.001
ALB	2.9 (2.5–3.3)	3.0 (2.6–3.4)	2.6 (2.2–3.1)	<0.001
TBil	1.1 (0.6–2.9)	1.1 (0.6–2.7)	1.0 (0.4–3.9)	0.54
ALT	54 (25–147)	55 (25–152.5)	50 (25–97)	0.43
AST	69 (34–177)	62 (34–172.5)	89 (36–181)	0.37
GLU	133 (105–175)	129 (105–169)	148 (104–206)	0.03
Crea	1.0 (0.7–1.7)	0.9 (0.7–1.3)	3.2 (1.8–5.2)	<0.001
BUN	21 (12–35)	17 (11–26)	63.5 (51–85)	<0.001
PT	14.4 (12.9–16.7)	14.3 (12.9–16.3)	15.0 (13.2–19.8)	0.01
INR	1.3 (1.2–1.5)	1.3 (1.2–1.5)	1.3 (1.2–1.8)	0.02
K	4.0 (3.6–4.6)	4.0 (3.6–4.4)	4.4 (4.0–5.0)	<0.001
Na	138 (135–141)	138 (135–141)	138.5 (133–143.5)	0.97
TCa	7.9 (7.2–8.4)	7.9 (7.3–8.4)	7.6 (6.9–8.45)	0.02
LAC	1.8 (1.2–2.8)	1.8 (1.2–2.8)	1.9 (1.3–2.8)	0.17
Blood lipase	232 (61–1,223)	214 (59–1,144)	286 (72–1,501)	0.19
Treatment				
Vasopressin, n (%)	97 (17.05)	64 (13.65)	33 (33.00)	<0.001
Octreotide, n (%)	50 (8.79)	35 (7.463)	15 (15.00)	0.02
Statins, n (%)	304 (53.43)	249 (53.09)	55 (55.00)	0.73
Betablockers, n (%)	505 (88.75)	417 (88.91)	88 (88.00)	0.79
Ventilation, n (%)	76 (13.36)	46 (9.81)	30 (30.00)	<0.001
CRRT, n (%)	38 (6.68)	34 (7.25)	4 (4.00)	0.24
ERCP, n (%)	97 (17.05)	64 (13.65)	33 (33.00)	<0.001
Clinical Outcomes				
14-day ACM, n (%)	46 (8.08)	25 (5.33)	21 (21.00)	<0.001
28-day ACM, n (%)	69 (12.13)	37 (7.89)	32 (32.00)	<0.001
90-day ACM, n (%)	107 (18.80)	64 (13.65)	43 (43.00)	<0.001
365-day ACM, n (%)	134 (23.55)	86 (18.34)	48 (48.00)	<0.001

BAR, blood urea nitrogen to albumin ratio; SBP, systolic blood pressure; DBP, diastolic blood pressure; MAP, mean arterial pressure; RR, respiratory rate; RBC, red blood cell; WBC, white blood cell; RDW, red blood cell distribution width; PLT, platelet; Hb, hemoglobin; HCT, hematocrit; AG, anion gap; ALB, albumin; TBil, total bilirubin; ALT, alanine aminotransferase; AST, aspartate aminotransferase; GLU, Glucose; Crea, creatinine; BUN, blood urea nitrogen; PT, prothrombin time; INR, international normalized ratio; K, serum potassium; Na, serum sodium; TCa, serum total calcium; LAC, lactate; CRRT, continuous renal replacement therapy; ERCP, endoscopic retrograde cholangiopancreatography; ACM, all-cause mortality.

### 3.2 The K–M curve analysis

**Figure 2:**
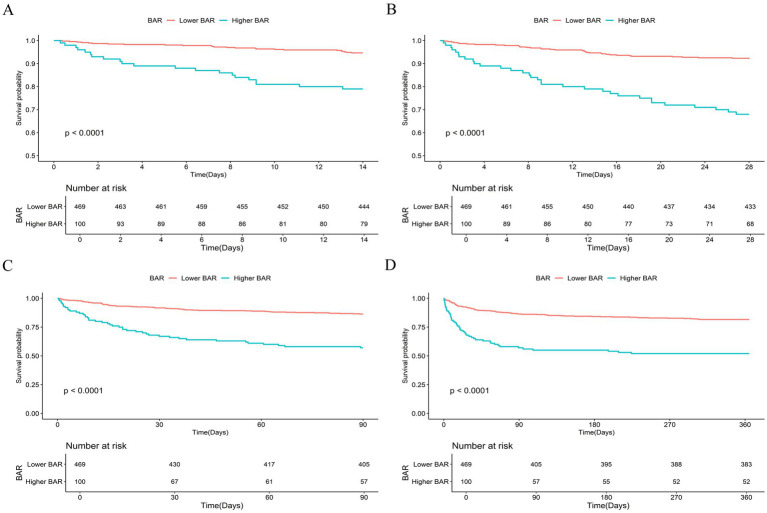
Kaplan–Meier curves and cumulative incidence of 14-day **(A)**, 28-day **(B)**, 90-day **(C)**, and 365-day **(D)** all-cause mortality stratified by BAR groups.

In this study, 46 of the 569 patients with AP died within 14 days, 69 died within 28 days, 107 died within 90 days, and 134 died within 1 year. The K–M curves demonstrated notable disparities in ACM between the two groups at days 14, 28, 90, and 1 year (Figure 2). At these time points, patients in the higher BAR group exhibited notably higher ACM compared to those in the lower BAR group (all log-rank *p* < 0.001).

### 3.3 Association between ACM and BAR

By constructing multivariate Cox regression models, we further evaluated the association between BAR and 14-day, 28-day, 90-day, and 1-year ACM among patients diagnosed with AP. In unadjusted Model 1, a higher BAR correlated significantly with an elevated risk of mortality over time, as follows: day 14 (HR, 4.37; 95% CI, 2.44–7.80; *p* < 0.001), day 28 (HR, 4.69; 95% CI, 2.92–7.53; *p* < 0.001), day 90 (HR, 3.92; 95% CI, 2.66–5.78; *p* < 0.001), and 1 year (HR, 3.41; 95% CI, 2.39–4.85; *p* < 0.001). Model 2 was adjusted for age, sex, and ethnicity. The findings indicated that individuals with elevated BAR continued to exhibit heightened mortality risks, as follows: day 14 (HR, 3.27; 95% CI, 1.81–5.90; *p* < 0.001), day 28 (HR, 3.67; 95% CI, 2.26–5.96; *p* < 0.001), day 90 (HR, 3.12; 95% CI, 2.10–4.64; *p* < 0.001), and 1 year (HR, 2.72; 95% CI, 1.90–3.9; *p* < 0.001). Model 3 was adjusted for additional potential confounders, including age, sex, ethnicity, hypertension, diabetes, heart failure, RF, sepsis, white blood cell count, Crea level, platelet count, vasopressin level, CRRT, and SOFA score. The results similarly confirmed that patients with higher BAR were still at a higher risk of ACM at days 14, 28, 90, and 1 year. Table 3 provides the detailed results.

**Table 3:** Cox proportional hazard ratios (HR) for all-cause mortality.

	Model 1		Model 2		Model 3	
	HR (95% CI)	*p* value	HR (95% CI)	*p* value	HR (95% CI)	*p* value
14-day ACM						
BAR (continuous)	1.07 (1.05–1.09)	<0.001	1.06 (1.04–1.08)	<0.001	1.08 (1.05–1.12)	<0.001
Lower BAR	Reference		Reference		Reference	
Higher BAR	4.37 (2.44–7.80)	<0.001	3.27 (1.81–5.90)	<0.001	3.06 (1.45–6.45)	<0.001
28-day ACM						
BAR (continuous)	1.06 (1.05–1.08)	<0.001	1.06 (1.04–1.08)	<0.001	1.07 (1.05–1.10)	<0.001
Lower BAR	Reference		Reference		Reference	
Higher BAR	4.69 (2.92–7.53)	<0.001	3.67 (2.26–5.96)	<0.001	3.16 (1.71–5.82)	<0.001
90-day ACM						
BAR (continuous)	1.06 (1.04–1.07)	<0.001	1.05 (1.04–1.06)	<0.001	1.06 (1.04–1.08)	<0.001
Lower BAR	Reference		Reference		Reference	
Higher BAR	3.92 (2.66–5.78)	<0.001	3.12 (2.10–4.64)	<0.001	2.54 (1.55–4.18)	<0.001
1-year ACM						
BAR (continuous)	1.05 (1.04–1.07)	<0.001	1.05 (1.03–1.06)	<0.001	1.06 (1.03–1.08)	<0.001
Lower BAR	Reference		Reference		Reference	
Higher BAR	3.41 (2.39–4.85)	<0.001	2.72 (1.90–3.90)	<0.001	2.24 (1.43–3.50)	<0.001

Model 1: Unadjusted; Model 2: Adjusted age, gender, and ethnicity; Model 3: Adjusted age, gender, ethnicity, Sequential Organ Failure Assessment, creatinine, red blood cell, platelet, sepsis, hypertension, heart failure, respiratory failure, diabetes, vasopressin, continuous renal replacement therapy.

### 3.4 Non-linear relationship detection

**Figure 3:**
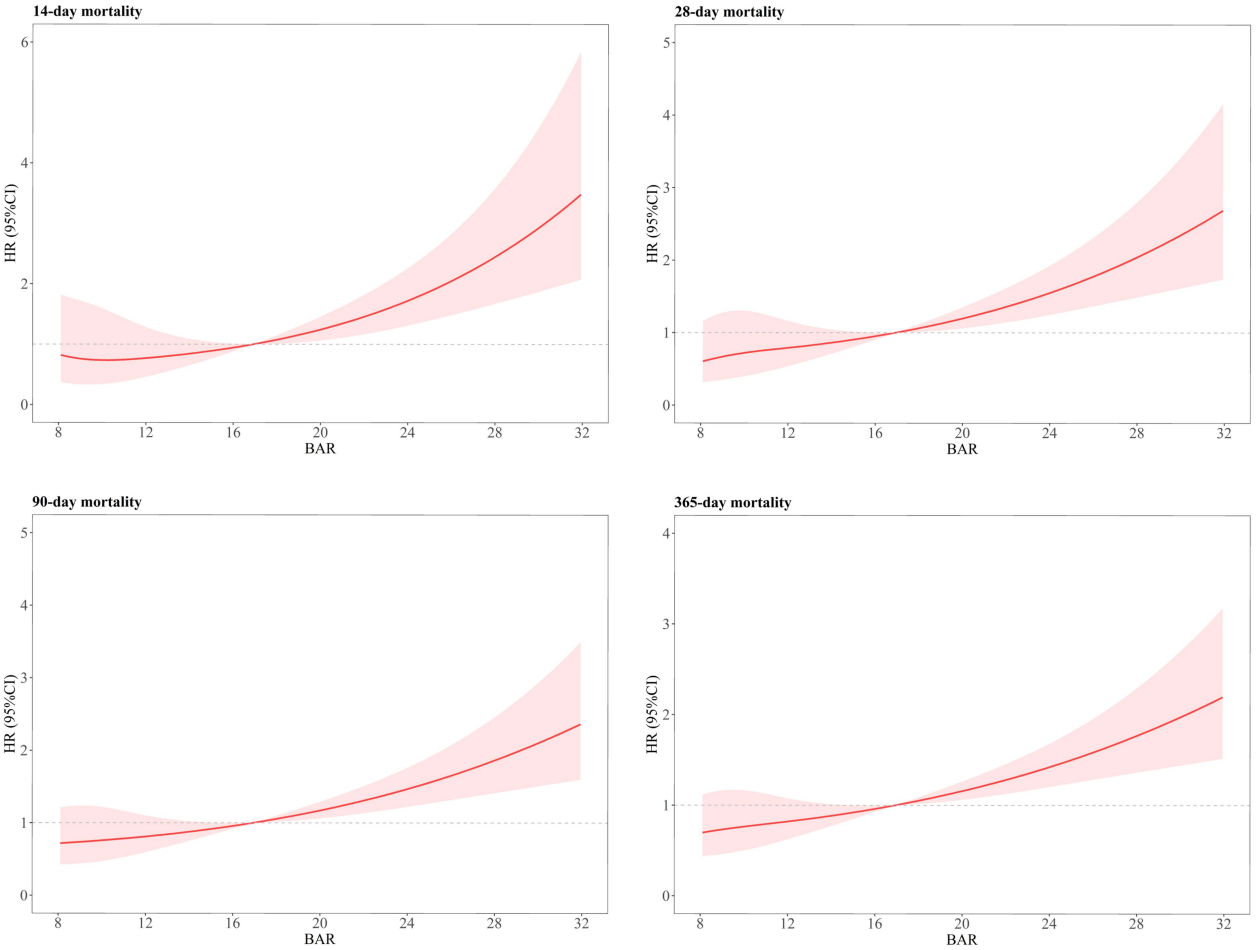
Restricted cubic spline curves of BAR with all-cause mortality in patients with acute pancreatitis.

RCS analyses revealed a J-shaped correlation between the BAR and ACM at days 14, 28, 90, and 1 year in AP patients (Figure 3). Specifically, the mortality risk among patients with AP exhibited a gradual increase when the BAR was <16.92; after exceeding 16.92, the risk of mortality increased significantly and rapidly (*P* for non-linear = 0.52 at day 14, *P* for non-linear = 0.21 at day 28, *P* for non-linear = 0.39 at day 90, and *P* for non-linear = 0.46 at 1-year).

### 3.5 Forecasting ACM in AP patients using BAR

**Figure 4:**
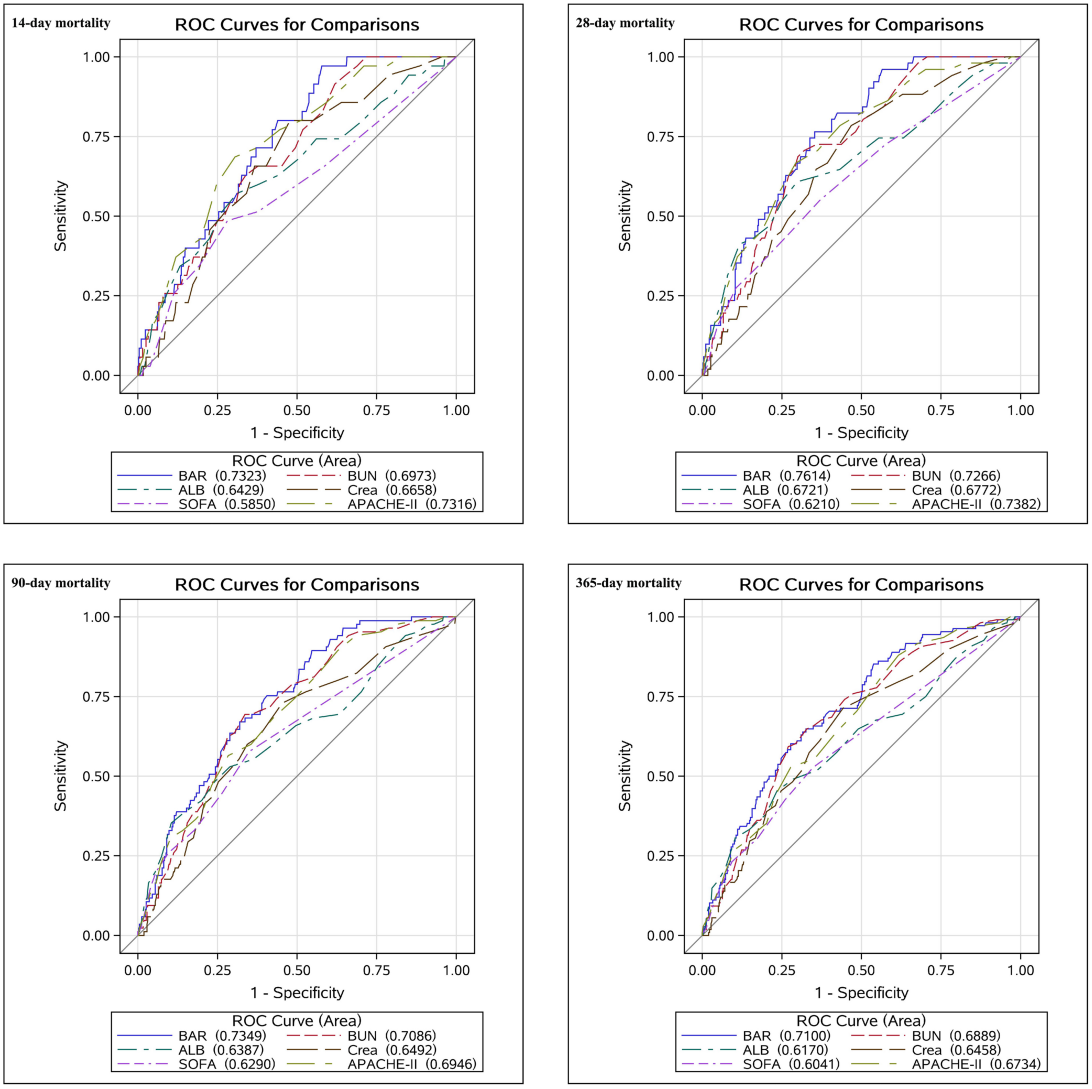
Receiver operating characteristic curves for predicting all-cause mortality in patients with acute pancreatitis.

By plotting ROC curves for BAR, BUN, ALB, Crea, SOFA, and APACHE-II, we evaluated their predictive values for ACM in patients at days 14, 28, 90, and 1 year. The results are shown in Figure 4. The results of our study revealed that BAR was superior to BUN, ALB, Crea, SOFA, and APACHE-II in predicting ACM at days 14, 28, 90, and 1 year in patients with AP in terms of AUC values. For example, compared with BUN [69.73% (95% CI, 61.88–77.57)], ALB [64.29% (95% CI, 54.07–74.50)], Crea [66.58% (95% CI, 58.05–75.11)], SOFA [58.50% (95% CI, 48.11–68.90)], and APACHE-II [73.16% (95% CI, 65.23–81.09)], BAR had a significantly improved AUC on day 14 [73.23% (95% CI, 66.18–80.28)]. In addition to this example, more detailed results are shown in Figure 4. The data depicted in Figure 4 are summarized in Table 4. Our findings highlight the exceptional predictive capacity of BAR for ACM among AP patients, highlighting its significant clinical utility.

**Table 4:** Information of ROC curves in Figure 4.

Variables	AUC (%)	95% CI (%)	Threshold	Sensitivity	Septicity
14-day mortality					
BAR	73.23	66.18–80.28	7.46	0.85	0.55
BUN	69.73	61.88–77.57	25.50	0.72	0.64
ALB	64.29	54.07–74.50	2.55	0.50	0.73
CR	66.58	58.05–75.11	1.05	0.85	0.48
SOFA	58.50	48.11–68.90	2.50	0.52	0.70
APACHE-II	73.16	65.23–81.09	14.50	0.68	0.76
28-day mortality					
BAR	76.14	70.35–81.93	8.48	0.78	0.63
BUN	72.66	66.37–78.94	26.50	0.74	0.69
ALB	67.21	58.60–75.82	2.55	0.49	0.74
CR	67.72	60.71–74.73	1.05	0.84	0.49
SOFA	62.10	53.73–70.47	4.50	0.36	0.88
APACHE-II	73.82	66.96–80.67	14.50	0.69	0.71
90-day mortality					
BAR	73.49	68.27–78.71	9.36	0.66	0.70
BUN	70.86	65.34–76.39	24.50	0.65	0.71
ALB	63.87	56.89–70.84	2.55	0.46	0.76
CR	64.92	58.54–71.30	1.05	0.78	0.50
SOFA	62.90	56.25–69.56	1.50	0.63	0.64
APACHE-II	69.46	63.71–75.21	14.50	0.70	0.60
365-day mortality					
BAR	71.00	65.72–76.28	9.36	0.63	0.71
BUN	68.89	63.46–74.33	26.50	0.63	0.72
ALB	61.70	55.34–68.07	2.55	0.43	0.76
CR	64.58	58.78–70.37	1.05	0.76	0.52
SOFA	60.41	54.33–66.50	1.50	0.58	0.64
APACHE-II	67.34	61.90–72.78	14.50	0.70	0.56

### 3.6 Subgroup analysis

**Figure 5:**
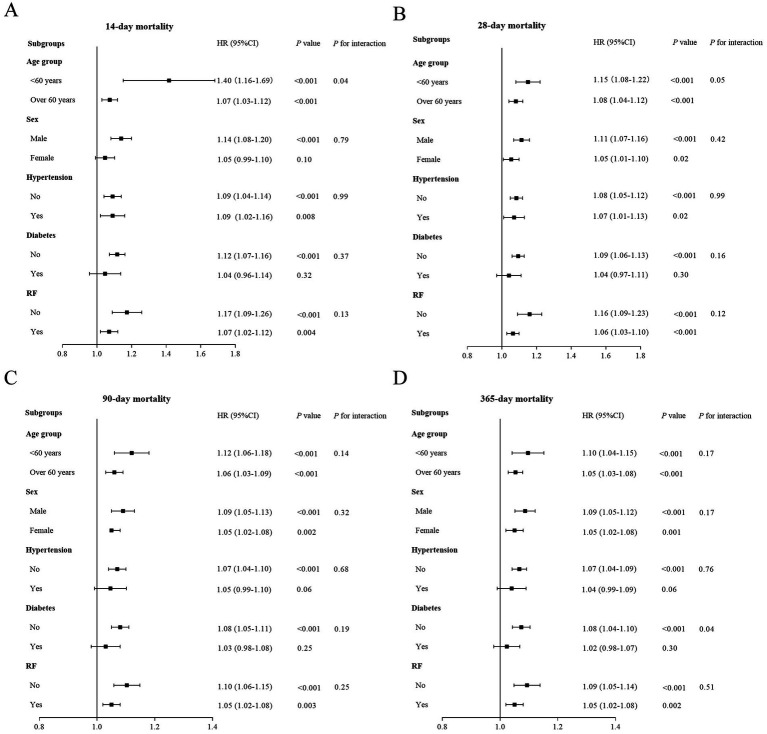
Forest plots of stratified analyses of BAR and 14-day **(A)**, 28-day **(B)**, 90-day **(C)**, and 365-day **(D)** all-cause mortality.

We further investigated the correlation between BAR and ACM at days 14, 28, 90, and 1 year across various subgroups of AP patients. When stratified by age, sex, hypertension, diabetes mellitus, and RF, the forest plots revealed non-significant interactions between BAR and the majority of subgroups (*p* > 0.05). Except for the day 14, we observed a minor interaction between BAR and age subgroups (*p* = 0.04), and at 1-year, a minor interaction was observed between BAR and diabetes subgroups (*p* = 0.04). More detailed results are presented in Figure 5. These results confirmed the robustness of our conclusions.

## 4 Discussion

AP is a gastrointestinal disorder characterized by elevated morbidity and mortality rates, particularly in patients admitted to the ICU. Despite improvements in intensive care management, prognostic markers of mortality in patients with AP continue to be explored. In this retrospective cohort analysis, we explored the correlation between BAR and ACM among ICU patients diagnosed with AP. Our findings revealed that elevated BAR levels independently correlated with an increased risk of ACM at 14-day, 28-day, 90-day, and 1-year post-admission. This relationship remained unchanged even after adjusting for potential confounders. The K–M survival analyses demonstrated a notable distinction: patients diagnosed with AP exhibiting BAR exceeding 16.92 exhibited considerably elevated levels of ACM at days 14, 28, 90, and 1 year compared to those with BAR of 16.92 or lower. Additionally, the BAR served as a reliable predictor of ACM among patients with AP. The AUC values demonstrated that the BAR had higher accuracy than BUN, ALB, Crea, SOFA, and APACHE-II. The subgroup analyses further confirmed the robustness of our conclusions. Hence, this study introduces a new, straightforward, and efficient biomarker for assessing mortality risk in AP patients.

BUN is a nitrogen-containing compound in the plasma that can be used to assess not only renal function but also the body’s nutritional status, blood volume, and protein metabolism (16). The BUN levels are often elevated in critically ill patients (e.g., those with acute heart failure and diabetes) (17, 18). Dai et al. (19) found that elevated BUN levels within 24 h of admission were independently associated with a higher risk of 30-day ACM in individuals diagnosed with AP. Our results further confirmed the significant increase in BUN levels in AP patients hospitalized in the ICU. Elevated BUN in AP patients is a multifactorial process that is closely related to the systemic inflammatory response and organ dysfunction that characterize severe AP (20). One of the main mechanisms is reduced renal perfusion due to hypovolemia and shock, which often accompany AP patients managed in ICU (21, 22). Hypovolemia may result from fluid loss into the third space, capillary leakage syndrome, or intravascular volume depletion, which leads to a decrease in glomerular filtration rate (GFR) and the subsequent accumulation of nitrogenous waste products such as urea in the blood (21, 22). In addition, a systemic inflammatory response characterized by elevated levels of cytokines, such as tumor necrosis factor-*α* (TNF-α) and interleukin-6 (IL-6), exacerbates renal dysfunction by promoting vascular permeability and fluid shifts, leading to prerenal azotemia (23, 24). Furthermore, during the entire pathological course, the pancreas releases large amounts of digestive enzymes that can damage the surrounding tissues and blood vessels. The body responds to tissue damage by synthesizing and releasing additional proteins as raw materials for repair and regeneration. The decomposition of these additional proteins increases the production of BUN, resulting in elevated BUN levels in the blood (25). Next, patients may experience symptoms such as vomiting and diarrhea, leading to fluid loss and dehydration, and the insufficient blood volume further activates the renin–angiotensin–aldosterone system to increase the reabsorption of BUN in the body (26–28). Moreover, patients with AP may simultaneously develop AKI. When the kidneys do not function properly, BUN excretion is reduced, leading to higher blood BUN levels (29, 30).

ALB serves not only as an indicator of the body’s nutritional status but also performs crucial functions in maintaining intravascular colloid osmolality and exerting anti-inflammatory and antioxidant effects (31). ALB is also commonly used to evaluate the severity of AP and the efficacy of treatment (32). Patients with AP are often hypoproteinemic, which is the result of a combination of decreased synthesis of ALB, increased catabolism, and redistribution of ALB due to inflammatory responses and altered vascular permeability (33). First, during the acute phase of pancreatitis, ALB production is frequently suppressed as the liver shifts to synthesize acute-phase proteins (e.g., C-reactive protein (CRP)) due to a systemic inflammatory response driven by cytokines (e.g., IL-6) (34, 35). At the same time, the increased vascular permeability resulting from the inflammatory cascade causes leakage of ALB into the interstitial space, leading to a decrease in circulating serum ALB (27, 34, 36). The development of AP is intricately linked to oxidative stress. In addition, inflammatory response activation and inflammatory cell recruitment lead to tissue damage, and ALB increases the generation of anti-inflammatory agents (such as lipoxins, hemopexins, and protective proteins), facilitating the restoration of injured tissue (32). This depletion of ALB is attributed to substantial utilization during this process, which could elucidate the frequently observed low ALB levels in AP patients. Moreover, AP often triggers a hypermetabolic state that leads to increased proteolysis, further reducing serum albumin levels. The combination of decreased synthesis, increased extravasation, and increased catabolism results in hypoalbuminemia, which in turn is associated with a worse clinical prognosis and higher mortality in patients with AP.

BAR integrates the clinical values of BUN and ALB for AP and is a comprehensive parameter that reflects renal function, liver function, inflammation, nutritional status, endothelial function, and blood volume. In recent studies, the BAR has been used to assess the prognoses of critically ill patients. Zhang et al. (37) found that an increased BAR predicted mortality in ICU patients with severe coronary artery disease. The AUC of BAR in predicting hospitalization, 28-day, and 1-year mortality were 0.671, 0.673, and 0.685, respectively, and its predictive performance was better than that of BUN or ALB alone. In another cohort study including 12,125 patients, Shi et al. (38) found a significant association between elevated BAR levels and heightened ACM among patients with AKI. Meanwhile, the ROC curve results suggested that BAR predicted 28-day and 365-day ACM, with an AUC of 0.649 and 0.662, respectively, which were superior to those of BUN and SOFA. Similarly, Nam et al. (39) observed a robust correlation between BAR and various types of cerebral small-vessel disease in individuals undergoing health screening. BAR is a more reliable predictor than BUN or ALB alone. In our study, the relationship between BAR and short- and long-term ACM in AP patients was influenced by the interplay between renal function, systemic inflammation, and nutritional status. BAR effectively integrates these two key metrics: serum BUN, which reflects the severity of renal impairment and systemic inflammatory response, and ALB, which reflects the nutritional and hepatic functional status of the patient. A higher BAR indicates elevated BUN and lower ALB, both of which are associated with a worse prognosis in critically ill AP patients. BAR is a reliable prognostic tool because it captures the cumulative burden of systemic inflammation, organ dysfunction, and malnutrition in patients with AP, which explains its strong association with short- and long-term mortality.

Similar results have been obtained by our research team. ACM significantly increased in AP patients at days 14, 28, 90, and 1 year when the BAR was >16.92. The ROC curves demonstrated that the AUC values of the BAR were higher than those of ALB, BUN, Crea, SOFA, and APACHE-II. While SOFA is frequently used as a scoring system for ICU patients and demonstrates favorable predictive efficacy for mortality in critically ill individuals (40, 41), our investigation revealed that BAR exhibited superior AUC values compared to SOFA in forecasting ACM among AP patients at days 14, 28, 90, and 1 year. APACHE-II is also one of the most widely used critical illness evaluation systems in the current ICU, which can objectively assess the severity of the patient’s condition and provide a scientific basis for the rational utilization of medical resources and the improvement of medical quality (1). Meanwhile, APACHE-II is also a commonly used prognostic evaluation tool for AP patients. According to our results, we believe that BAR has an advantage over more complex scoring systems (e.g., SOFA or APACHE-II) in evaluating the prognosis of AP (especially critically ill patients hospitalized in ICU). Although these scoring systems are valuable, they typically require the integration of multiple clinical variables and are not readily available for use in all clinical settings. For example, the APACHE-II scoring system consists of three main components: the Acute Physiology Score (APS), the Age Score, and the Chronic Health Score (CHS). The APS, in turn, contains 12 physiologic indicators, and the CHS contains 5 components. Thus, the APACHE-II is complicated to calculate. In contrast, the BAR provides a simple, readily available, and cost-effective tool that can be calculated using two routine laboratory measurements (BUN and ALB), making it a practical option for clinicians in both resource-rich and resource-limited settings. More importantly, our study demonstrated that BAR has a higher predictive accuracy compared to BUN, ALB, Crea, SOFA, and APACHE-II, as reflected by the AUC values for short- and long-term ACM. This finding emphasizes the potential clinical utility of BAR as an independent and easily implemented prognostic marker for patients with AP in the ICU.

A major advantage of this study is that it is the first to propose that BAR is an independent predictor of short- and long-term ACM in ICU-managed AP patients. The extensive and diverse population data in the MIMIC-IV database allowed us to make comprehensive statistical adjustments, effectively control for potential confounding variables, and ensure the reliability of our findings. Our findings have important clinical implications. Early assessment of the BAR can help clinicians identify patients at high risk for poor outcomes in AP and enable timely interventions to improve prognosis. Compared with other complex scoring systems, the BAR has the advantage of being simple, economical, and easy to calculate, and it can be used in a variety of healthcare settings, including resource-limited healthcare organizations.

While our study offers valuable insights into the prognostic significance of the BAR in AP patients, it is important to acknowledge several limitations. First, our study was limited by its retrospective nature and single-center design, which may restrict the generalizability of our findings. Therefore, prospective multicenter studies are warranted. Second, due to constraints inherent to the MIMIC-IV database, the precise causes of mortality among patients with AP remain indeterminate, thereby constraining our capacity to evaluate the BAR’s prognostic utility in AP-specific mortality. The inclusion of detailed etiological data is necessary for future studies. Third, our analyses focused on initial BAR levels at the time of patient admission; therefore, the dynamic alterations in BAR over time were beyond our scope of assessment. Future studies on the predictive value of dynamic BAR measurements are required to elucidate their clinical utility. In addition, malnutrition has a significant impact on serum BUN and ALB levels. However, we were unable to assess the extent to which prehospital malnutrition affects the predictive value of BAR in AP patients hospitalized in the ICU because of the inability to know the nutritional level of the patients before admission. Therefore, future studies are necessary to further explore the potential impact of nutritional status on the predictive value of BAR. Finally, despite multivariate adjustments and subgroup analyses, several other potential confounders may have affected the results.

## 5 Conclusion

Our research revealed a notable correlation between BAR and ACM among ICU patients with AP. The BAR stands out as a promising prognostic biomarker, offering clinicians a straightforward and efficient tool to stratify risk and promptly identify patients with a heightened risk of mortality. Future prospective studies are necessary to validate our findings and elucidate the intrinsic association between BAR and AP mortality outcomes.

## Data Availability

The original contributions presented in the study are included in the article/Supplementary material; further inquiries can be directed to the corresponding authors.

## Glossary

AKI: acute kidney injury
ALB: albumin
ANOVA: analysis of variance
AP: acute pancreatitis
AUC: area under the curve
BAR: blood urea nitrogen to albumin ratio
BUN: blood urea nitrogen
CI: confidence interval
Crea: creatinine
HR: hazard ratio
ICD-10: International Classification of Diseases, Revision 10
ICU: intensive care unit
IQR: interquartile range
MIMIC-IV: Medical Information Mart for Intensive Care-IV
RF: respiratory failure
K–M: Kaplan–Meier
ROC: receiver operating characteristic
RCS: restricted cubic spline
SD: standard deviation
SOFA: Sequential Organ Failure Assessment
SQL: Structured Query Language
CRRT: continuous renal replacement therapy
APACHE-II: Acute Physiology and Chronic Health Evaluation-II.
