# Supplementary figures and images for: Exploring the value of blood urea nitrogen-to-albumin ratio in patients with acute pancreatitis admitted to the intensive care unit: a retrospective cohort study
Source: Front Nutr. 2025 Apr 16;12:1435356. doi: 10.3389/fnut.2025.1435356 (PMC12040672; doi:10.3389/fnut.2025.1435356)

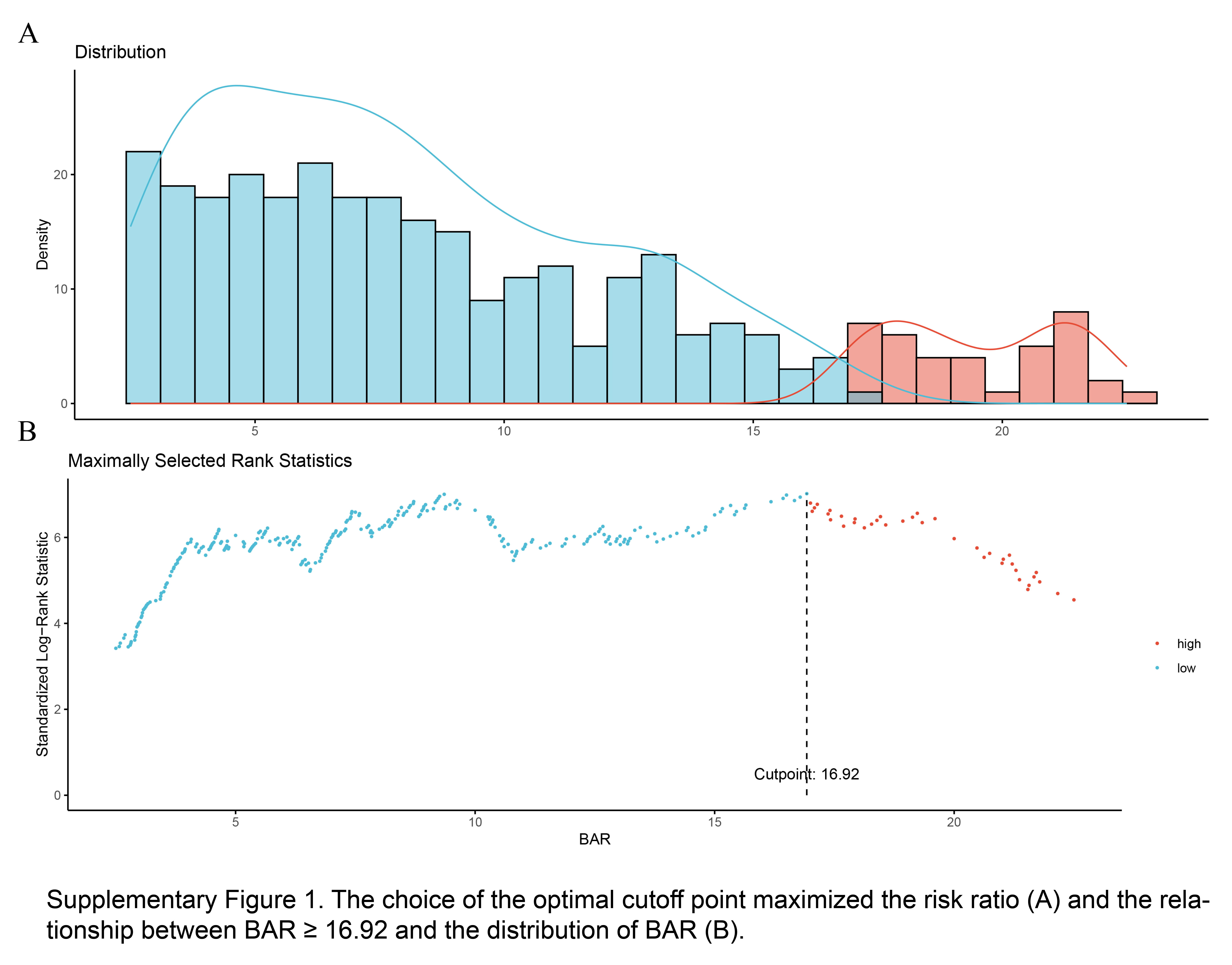

Supplement: Supplementary file 1 [file Image_1.jpg]
